# Editorial: Recent trends in nanotechnology in precision and sustainable agriculture

**DOI:** 10.3389/fpls.2023.1256319

**Published:** 2023-08-09

**Authors:** Pankaj Kumar Tyagi, Arvind Arya, Seema Ramniwas, Shruti Tyagi

**Affiliations:** ^1^ Department of Biotechnology, Noida Institute of Engineering and Technology, Greater Noida, India; ^2^ University Centre for Research and Development, Chandigarh University, Mohali, India; ^3^ WOS-B, Scientist of DST-Scheme, Noida Institute of Engineering and Technology, Greater Noida, India

**Keywords:** anotechnology, nanomaterials, nanofertilisers, precision farming, sustainable agriculture

The goal of this research on contemporary advancements in nanotechnology for precision and sustainable agriculture is to improve agricultural practices by making use of the special qualities of nanomaterials, nanoparticles, and nano devices. Nanotechnology provides creative solutions for the problems encountered by agriculture by increasing precision in monitoring soil conditions and insect infestations, optimising resource utilisation, and minimising environmental effects (Tyagi et al., 2021; Yadav et al., 2023) Furthermore, nanotechnology can help in the development of crop types that are resilient to stress, allowing farmers to adjust to climate change and guarantee long-term sustainability in food production. The goal of the investigation of nanotechnology in agriculture is to develop farming methods that are more robust, ecologically friendly, and effective. In conventional farming, over one-third of crops are lost due to insect infestation, microbial attacks, natural disasters, poor soil quality, and decreased nutrient availability. To solve these problems, novel technologies are required (Haris et al., 2023). The agro technological revolution and sustainable agriculture, which have the potential to change the agricultural system and provide food security, have been made possible by precision nanotechnology. The indiscriminate use of pesticides and chemical fertilisers, which diminished soil biodiversity and created resistance to diseases and pests, was a problem that the green revolution helped to address. For precision farming, improved biosensors may be made using nanomaterials, nanoparticles, and nanochips to transport ingredients to plants. Traditional fertilisers, pesticides, and herbicides that have been nano-encapsulated aid in the delayed and sustained release of nutrients and agrochemicals, giving the plants precise amounts. Plant growth regulators and immune system enhancers are two recent commercial applications of nano fertilisers (Zaim et al., 2023). They contain essential elements (such as iron, titanium dioxide, silica, and zinc) that play critical roles in plant growth, nutrient absorption, and disease resistance. Their presence in the soil ensures that crops receive the nutrients they require for optimal growth and yield. Pests frequently infest agricultural fields and reduce agricultural productivity. The development of nano-encapsulated insecticides has reduced the pesticide dosage. Therefore, the development of non-toxic and potential pesticide delivery methods has contributed to both an increase in world food production and a reduction in the negative environmental consequences of pesticides. Although nanotechnology offers numerous advantages, it also runs a risk of causing damage. Metal nanoparticles, for instance, may be harmful depending on the charge at the membrane surface. Nanoparticles enter the cellular system of plants through the shoot, root, and leaf systems. In order to survive, these nanoparticles aggregate throughout the aerial parts of plants and respond to different levels of trophic balance. It is unclear if the accumulation of nanoparticles in the plant system slows down transpiration, respiration, photosynthesis, and food transport or if these physiological processes are unaffected. The primary objective of giving a cross-section of current modern nanotechnology developments as they relate to precision and sustainable agriculture has been achieved by the research articles published in this field of study.

Iqbal et al. develop a protocol for nanotechnology-based elicitor-assisted *in vitro* shoot multiplication and callus induction of different mung bean varieties, aiming to enhance their phytochemical content. Shoot tips and nodal tips from three mung bean varieties were utilized as explants for shoot multiplication. Both *in vitro* and *in vivo* explants were cultured on MS media supplemented with varying concentrations of BAP and IBA as individual treatments. On the other hand, calli were cultivated on MS medium supplemented with zinc oxide and copper oxide nanoparticles as nano-elicitors to stimulate the production of phenolic and glycosides. The results demonstrated that *in vitro* explants outperformed *in vivo* explants in terms of shoot length, number of shoots, and number of leaves per explant. Additionally, shoot tips displayed better responsiveness to *in vitro* culture conditions compared to nodal explants. It was suggested that using nanoparticles to elicit secondary metabolites from *in vitro* mung bean cells could be a successful approach.

Nitin et al. investigated the contemporary method for detecting pests of castor plants. Castor crop a significant non-edible industrial crop, that yields oil used in the production of lubricants, lubricating grease, and other products. However, the quantity and quality of castor oil can be negatively impacted by insect pest attacks. The conventional approach to identifying pests is time-consuming and requires extensive knowledge. To address this issue, the authors of this research propose the use of automated insect pest detection techniques and precision agriculture to support sustainable agriculture practices. The accuracy of the recognition system relies on real-world data, which may not always be readily available. Data augmentation is a common method used to enrich the dataset in such cases. As part of this study, an insect pest dataset consisting of typical castor pests was created. To overcome the challenge of insufficient data for effective training of vision-based models, this research suggests a hybrid manipulation-based technique for data augmentation. Subsequently, the deep convolutional neural networks VGG16, VGG19, and ResNet50 were employed to evaluate the performance of the proposed augmentation technique. The prediction results demonstrate that this approach significantly outperforms previous methods in terms of overall performance while addressing the challenges associated with dataset size.

Geremew et al. create zinc oxide nanoparticles from the leaf of the pecan (*Carya illinoinensis*) tree and investigate their role in the growth of mustard. Climate change and land degradation have an influence on the sustainability of food production, and the advanced use of nanotechnology is crucial to overcoming this difficulty. A foundation for the production of nano fertilizers and nanocomposite materials for larger agricultural uses and high-quality human nutrition might be provided by the production of nanomaterials based on important minerals like zinc. Therefore, the objective of this work was to create zinc oxide ZnONPs using the leaf extract of the *Carya illinoinensis* tree and examine how they affected mustard development, physiology, nutritional content, and antioxidant capabilities. Potential applications for ZnONPs include stimulating plant growth and improving crop yields through the use of a new soil supplement. In addition, ZnONP biofortification of *B. juncea* plants enhances the crop’s nutritional value and may enhance its therapeutic benefits.

Xu et al. explore the development of a detection model called ASFL-YOLOX for insect pests from the *Papilionidae family* in citrus orchards. The model addresses the need for a balance between speed and accuracy in pest detection. It incorporates various optimizations such as the Tanh-Softplus activation function, efficient channel attention mechanism, adaptive spatial feature fusion module, and soft DIoU non-maximum suppression algorithm. A structured pruning curation technique is also proposed to reduce unnecessary network parameters. Experimental results show that ASFL-YOLOX outperforms previous models in terms of speed and accuracy. It achieves a faster inference speed and higher mean average precision (mAP) compared to other models like YOLOv7-x, YOLOv7-tiny, SSD300, and Faster R-CNN. The model parameters of ASFL-YOLOX are compressed by 88.97%, reducing computational requirements while maintaining a mAP higher than 95%. The model is suitable for detecting pest stress in orchards and can be used in embedded systems, providing technical support for pest identification and localization systems in orchard plant protection equipment.

Ma et al. find the silver nanoparticles in cut tree peony flowers to improve quality and vase life. The researchers synthesized Ag-NPs using *Eucommia ulmoides* leaf extract and found that they inhibited bacterial growth. Treating the flowers with Ag-NPs increased flower size, weight, and water balance. The treated petals had lower levels of oxidative stress markers and higher levels of antioxidant enzymes. Furthermore, Ag-NPs reduced bacterial growth in the stem vessels. Overall, cut flowers were pre-treated with Ag-NPs in this technique, which improved water uptake, vase life, and flower quality. Nanotechnology implemented in the cultivation of cut tree peonies is a promising technique for the cut flower industry.

All these studies employed in recent trends in nanotechnology for precision and sustainable agriculture have shown great potential in improving crop productivity, resource utilization, and environmental impact. The application of nanotechnology in precise and sustainable agriculture is depicted diagrammatically in **Figure 1** along with current trends in the field.

**Figure 1:**
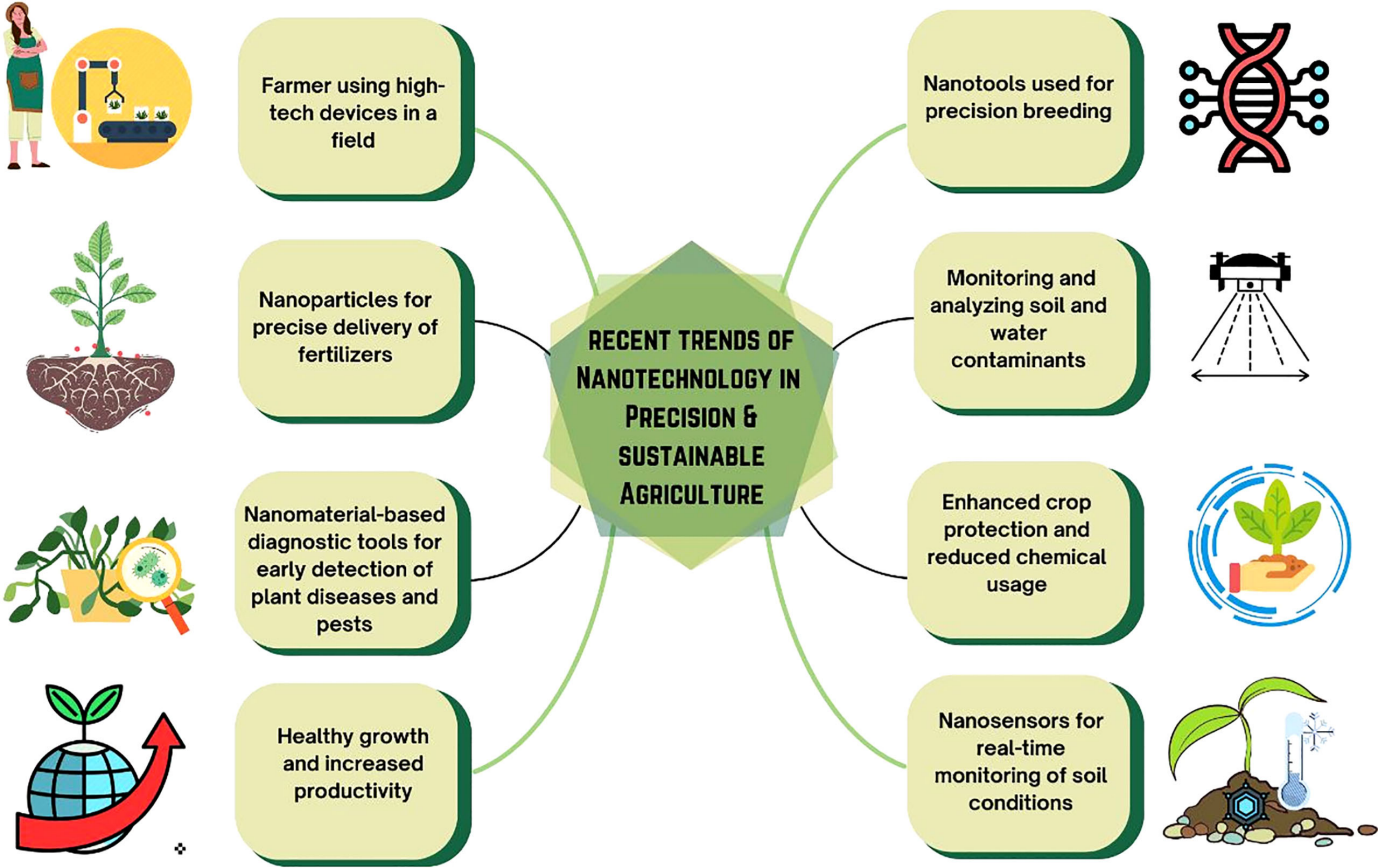
Recent trends of nanotechnology in precision agriculture.

Continuous monitoring of crops is made possible by nanosensors, nutrient absorption and pesticide delivery are improved by nanomaterials, and the breeding of crop types that can withstand stress is made easier by nanogenomics. However, for the safe and ethical application of nanotechnology in agriculture, thorough risk assessment and ethical considerations are essential. Overall, nanotechnology presents fascinating chances for an efficient and sustainable future of farming.

## Author contributions

PT: Conceptualization, Supervision, Writing – original draft, Writing – review & editing. AA: Validation, Writing – review & editing. SR: Conceptualization, Supervision, Writing – review & editing. ST: Formal Analysis, Visualization, Writing – review & editing.
